# Lipid oxidation controls peptide self-assembly near membranes through a surface attraction mechanism†

**DOI:** 10.1039/d3sc00159h

**Published:** 2023-03-02

**Authors:** Torsten John, Stefania Piantavigna, Tiara J. A. Dealey, Bernd Abel, Herre Jelger Risselada, Lisandra L. Martin

**Affiliations:** a School of Chemistry, Monash University Clayton VIC 3800 Australia tjohn@mit.edu Lisa.Martin@monash.edu; b Leibniz Institute of Surface Engineering (IOM) Permoserstraße 15 04318 Leipzig Germany; c Wilhelm-Ostwald-Institute for Physical and Theoretical Chemistry, Institute of Chemical Technology, Leipzig University Linnéstraße 3 04103 Leipzig Germany; d Institute for Theoretical Physics, Georg-August-Universität Göttingen Friedrich-Hund-Platz 1 37077 Göttingen Germany

## Abstract

The self-assembly of peptides into supramolecular structures has been linked to neurodegenerative diseases but has also been observed in functional roles. Peptides are physiologically exposed to crowded environments of biomacromolecules, and particularly cellular membrane lipids. Previous research has shown that membranes can both accelerate and inhibit peptide self-assembly. Here, we studied the impact of membrane models that mimic cellular oxidative stress and compared this to mammalian and bacterial membranes. Using molecular dynamics simulations and experiments, we propose a model that explains how changes in peptide-membrane binding, electrostatics, and peptide secondary structure stabilization determine the nature of peptide self-assembly. We explored the influence of zwitterionic (POPC), anionic (POPG) and oxidized (PazePC) phospholipids, as well as cholesterol, and mixtures thereof, on the self-assembly kinetics of the amyloid β (1–40) peptide (Aβ_40_), linked to Alzheimer's disease, and the amyloid-forming antimicrobial peptide uperin 3.5 (U3.5). We show that the presence of an oxidized lipid had similar effects on peptide self-assembly as the bacterial mimetic membrane. While Aβ_40_ fibril formation was accelerated, U3.5 aggregation was inhibited by the same lipids at the same peptide-to-lipid ratio. We attribute these findings and peptide-specific effects to differences in peptide-membrane adsorption with U3.5 being more strongly bound to the membrane surface and stabilized in an α-helical conformation compared to Aβ_40_. Different peptide-to-lipid ratios resulted in different effects. We found that electrostatic interactions are a primary driving force for peptide-membrane interaction, enabling us to propose a model for predicting how cellular changes might impact peptide self-assembly *in vivo*.

## Abstract 2

Oxidized model membranes have differential effects on peptide fibril formation, driven by surface attraction, peptide charge and secondary structure stabilization.

## Graphical abstract

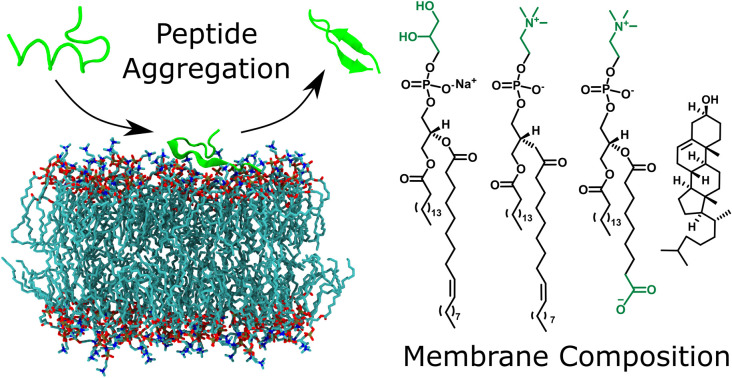

## Introduction

The self-assembly of peptides in a physiological environment into supramolecular structures such as fibrils has been implicated in ageing-related and neurodegenerative diseases.^1^ One example is amyloid β peptide (Aβ) that aggregates in the brains of patients diagnosed with Alzheimer's disease.^2,3^ However, peptide fibrils have not only been related to disease but were identified as functional, non-pathological states, and have developed structural advantages as functional materials.^4,5^ The fibril-forming peptide uperin 3.5 (U3.5) was first isolated as an antimicrobial peptide (AMP) and may be related to the innate immune system of the Australian toadlet *Uperoleia mjobergii*.^6–8^ Peptide fibrils are typically water-insoluble and form a common cross-β sheet structure, as observed by electron microscopy and X-ray diffraction.^9,10^ Recent cryoEM studies also identified cross-α fibril structures for a number of peptides.^11–14^

**Fig. 1:**
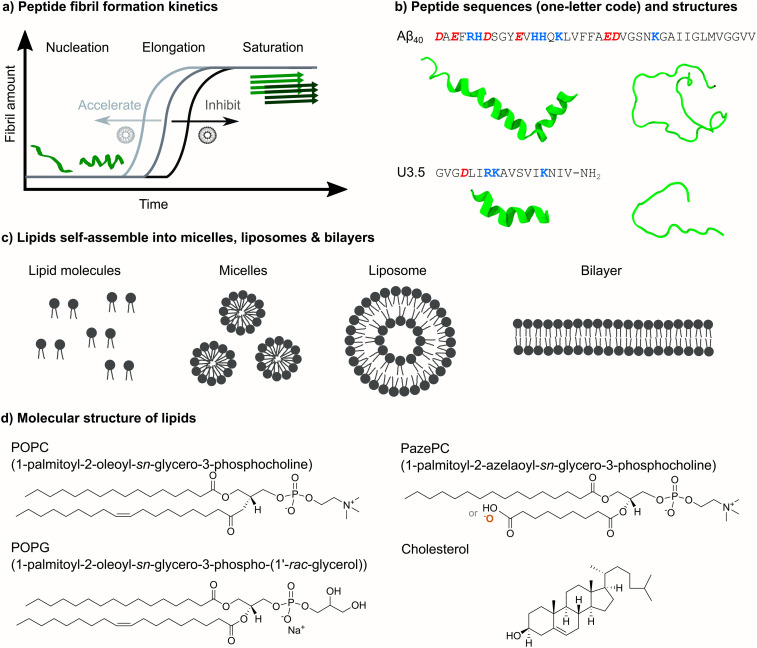
Overview of peptide self-assembly into fibrils and studied peptides and lipids. (a) Typical nucleation–elongation kinetics of fibril formation with the slow formation of critical nuclei and subsequent rapid fibril growth. The presence of lipids accelerates or inhibits peptide fibril formation, resulting in shorter or longer times for the nucleation phase, respectively. (b) Peptide sequences of Aβ_40_ and U3.5 in one-letter code (acidic groups: red and bold italics, basic groups: blue and bold) and α-helical and random coil secondary structures. (c) Phospholipids with hydrophilic head groups and hydrophobic tails typically spontaneously self-assemble into micelles, liposomes and lipid bilayers. (d) The chemical structure of the studied phospholipids POPC, POPG and PazePC as well as of cholesterol is shown. The carboxyl group of PazePC may be (partially) deprotonated under experimental conditions.

The formation of peptide fibrils follows typical nucleation-elongation kinetics with a slow nucleation phase followed by a fast elongation and growth of the peptide oligomers into fibrillar aggregates (Fig. 1a).^15,16^ Several studies suggested an α-helical peptide conformation as an intermediate towards β-sheet rich fibrils.^17–20^ However, the physiological role of amyloid-forming peptides and the biochemical processes that cause aggregation and disease are still under investigation.^21,22^ Since antimicrobial properties have not only been found for U3.5 but also for the Alzheimer-related Aβ peptide,^23^ studies suggested links between the antimicrobial activity of peptides and their connection to disease mechanisms.^8,23–25^

Under physiological conditions, peptides are surrounded by other biomacromolecules in crowded environments and cell membranes play an important role.^26–29^ This is particularly relevant as the pathology of amyloid-forming peptides has been linked to their peptide membrane activity.^30–32^ Both the impact of membranes on peptide structure and self-assembly kinetics as well as the action of peptides on membranes have been studied extensively.^33–43^

The membrane damage caused by amyloid-forming peptides has been attributed to different oligomeric species as well as the fibril growth process.^44,45^ Previous work either proposed the disruption of membranes by peptides or the modulation of peptide self-assembly by membranes as the initial process in relation to disease, or considered both processes as concomitant.^33,35,36,46,47^ Sparr and Linse emphasized the important role of membrane properties and particularly the protein-to-lipid ratio among the factors contributing to lipid–protein interactions in amyloid formation.^48^

In this work, we focused our attention on the role of the oxidized membrane lipid PazePC on the structure and self-assembly kinetics of the amyloid β (1–40) peptide (Aβ_40_)^2,3,49^ and the antimicrobial peptide uperin 3.5 (U3.5) (Fig. 1b),^6–8^ aggregating near membranes. While Aβ_40_ is a widely studied peptide related to Alzheimer's disease,^2^ U3.5 has originally been identified as an AMP.^6^ AMPs are generally cationic and known to adopt an α-helical conformation when in contact with membrane surfaces, stabilizing either intermediates towards peptide fibrils or off-pathway oligomers.^17,50–53^ Interestingly, the membrane disruption activity of antimicrobial and amyloid-forming peptides have been linked recently.^7,8,24,54^ There have also been reports on the relationship between fibril formation and environmental factors, such as oxidative stress and viral infections.^55–58^

Membranes constitute barriers and interfaces of complex composition and varying surface geometry.^40,59–62^ Along with sphingolipids, sterols and membrane proteins, phospholipids are the major components of membranes that self-assemble into micelles, liposomes and bilayers (Fig. 1c).^59^ Numerous studies identified a significant impact of membrane composition on peptide self-assembly, ranging from acceleration to inhibition of the process.^17,39,63–72^ Here, we studied membrane compositions consisting of zwitterionic 1-palmitoyl-2-oleoyl-*sn*-glycero-3-phosphocholine (POPC) as a major lipid bilayer component (Fig. 1d). Phosphatidylcholine (PC) lipids, such as POPC, are the main component of mammalian and bacterial cell membranes,^62^ and are typically used for biomimetic membrane studies.^73^ In addition to POPC, our model membranes and liposomes consisted of cholesterol, a typical component of mammalian membranes, as well as anionic 1-palmitoyl-2-oleoyl-*sn*-glycero-3-phospho-(1′-rac-glycerol) (POPG), a typical component of bacterial membranes.^8,74,75^ Furthermore, we studied the role of oxidative stress on peptide self-assembly by including the oxidized lipid 1-palmitoyl-2-azelaoyl-*sn*-glycero-3-phosphocholine (PazePC).^76–78^ Oxidative stress has previously been linked to ageing and neurodegenerative diseases.^79,80^ We used biophysical techniques to follow peptide self-assembly kinetics in the absence and presence of various lipid mixtures and peptide-to-lipid ratios to understand the impact on peptide secondary structure and peptide-membrane adsorption. Molecular dynamics (MD) simulations revealed molecular insights into the peptide-membrane interactions.

We observed differential effects of lipids on peptide self-assembly, depending on membrane composition and peptide-to-lipid ratio, with larger effects for the anionic POPG and the oxidized PazePC lipids, particularly for the U3.5 peptide. POPG and PazePC attracted the peptides onto their surface, driven by electrostatic interactions, and thereby influenced peptide secondary structure, leading to a large impact on peptide self-assembly.^81^ Interestingly, the same lipids and lipid mixtures, and peptide-to-lipid ratios led to differential effects for Aβ_40_ and U3.5 peptide, resulting from varying peptide-membrane attraction, hence secondary structure stabilization. Our results support the hypothesis that cellular changes, such as oxidative stress, trigger peptide self-assembly processes and could initiate or inhibit amyloid fibril formation, thus be related to the onset and progression of diseases.

## Results and discussion

To understand the influence of changes in the (cellular) membrane environment, namely the impact of an oxidized lipid, on the self-assembly of peptides into fibril structures, we initially performed experiments to follow the kinetics for the two peptides, amyloid β (1–40) (Aβ_40_) and uperin 3.5 (U3.5). We chose those peptides because of their similarity in forming fibrils as well as showing antimicrobial properties,^6,8,23,49^ but also their difference in overall charge, with Aβ_40_ being overall negatively charged and U3.5 positively charged (Table 1). As membranes, we used bilayers and liposomes or micelles (Fig. 1c) with the phospholipid POPC as the major component. Mixtures of POPC with cholesterol were used to model mammalian cells, mixtures of POPC with anionic POPG to mimic bacterial cells, and mixtures of POPC with PazePC to mimic oxidized membranes.^8,74,75^ PazePC has previously been identified as a major product of oxidative processes, and may be protonated or deprotonated at physiological pH (Table 1).^76–78^

**Table 1:** Charges of the peptides and lipids at pH 7.4

	Charge at pH 7.4	Charged residues
**Peptides**		
Aβ_40_	−2.9	6 × −1 (3 × Asp, 3 × Glu), 3 × +1 (Arg, 2 × Lys), +0.1 (His), -1 (C-terminus), +1 (N-terminus)
U3.5	+3	−1 (Asp), 3 × +1 (Arg, 2 × Lys), +1 (N-terminus)

**Lipids**		
POPC	±0	−1 (phosphate), +1 (choline)
Cholesterol	±0	Neutral
POPG	−1	−1 (phosphate)
PazePC	−1 to ±0	−1 (phosphate), +1 (choline), −1 (if azelaoyl carboxyl group is deprotonated)^78,82^

**Fig. 2:**
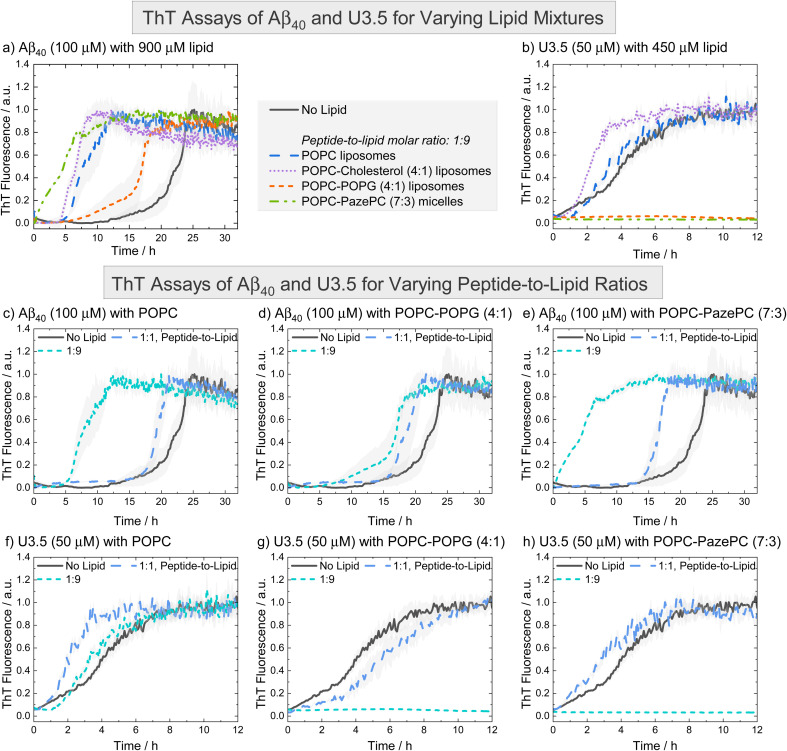
ThT fluorescence assays were performed to follow the kinetics of fibril formation. (a and b) The peptides Aβ_40_ (100 μM) and U3.5 (50 μM) were studied in PBS buffer at pH 7.4 at 37 °C. (c–h) Peptides were studied without and with different amounts of lipids present (peptide-to-lipid molar ratio: 1 : 1, 1 : 9). The largest impact of the lipids on peptide aggregation was observed when lipid was added in excess (1 : 9). When peptide and lipid had the same concentration in the sample (1 : 1), smaller effects were observed. Data for pure POPC and lipid mixtures consisting of POPC-POPG (4 : 1) and POPC-PazePC (7 : 3) are shown. Additional data can be found in ESI Fig. S3.† The data for U3.5 (b) without lipid present was previously reported and is included as a reference to all other lipids and Aβ_40_.^17^ The lines refer to the mean and the shadow areas to the SEM (standard error of the mean) of the replicates. Data were normalized to a maximum fluorescence of 1 (except in cases with inhibition of peptide aggregation).

To probe the influence of the oxidized lipid PazePC on peptide self-assembly, we used thioflavin T (ThT) fluorescence assays. ThT is a commonly used dye to detect peptide self-assembly into amyloid fibrils, as it shows an enhanced fluorescence upon binding to aggregates.^83,84^ We studied the peptides Aβ_40_ and U3.5 without and with different amounts of lipids present (Fig. 2). The peptide-to-lipid ratio was varied to study the situation with equal (molar) amounts of peptide and lipid (1 : 1) and excess of lipids (1 : 9). The lipids were added to the peptide solutions as liposomes, with the exception of POPC-PazePC and pure PazePC, which were present as micelles under the conditions used in this study, since PazePC has a relatively high critical micelle concentration (CMC) of ≈20 μM compared to POPC, POPG or cholesterol with CMC values in the nM range (see dynamic light scattering (DLS) measurements in ESI Fig. S2†).^82,85–88^ The fluorescence assays consistently showed the greatest effects for ratios where lipids were in excess (shown in Fig. 2, and S3, ESI † for additional lipids and lipid mixtures). While Aβ_40_ aggregation was accelerated in the presence of all lipids (*i.e.* shorter lag times) (Fig. 2a, c–e and S3a, c, e, g, ESI†), the aggregation of U3.5 was only minimally affected by POPC or cholesterol containing liposomes (POPC, cholesterol, POPC-cholesterol, 4 : 1) (Fig. 2b, f and S3b, h, ESI†). If POPG or PazePC lipids were present (POPG, PazePC, POPC-POPG, 4 : 1, and POPC-PazePC, 7 : 3) (Fig. 2b, g-h and S3d, f, ESI†), U3.5 aggregation was completely inhibited.

The acceleration of Aβ_40_ aggregation in the presence of membrane surfaces is in agreement with previous studies^89^ while an enhanced β-sheet formation of the related Aβ_42_ peptide has been observed on oxidatively damaged surfaces, in particular.^69^ The inhibition of U3.5 aggregation in the presence of POPG containing liposomes (POPG, POPC-POPG, 4 : 1) is in agreement with our prior work in which DMPG (1,2-dimyristoyl-*sn*-glycero-3-phospho-(1′-rac-glycerol)) containing liposomes were studied.^17^ In this work, we show that micelles containing the oxidized lipid PazePC have similar effects on peptide aggregation as POPG containing liposomes (Fig. 2h and S3f, ESI†). Clearly, Aβ_40_ and U3.5 peptide aggregation were influenced by anionic and oxidized lipids, albeit in a different direction, requiring us to consider the physicochemical properties of the peptides as illustrated above (Table 1). We will establish a model explaining the contrary effects on Aβ_40_ and U3.5 self-assembly, while discussing previous work by Kinnunen *et al.* and Axelsen *et al.* that also reported accelerating and inhibiting effects of oxidized lipids on amyloid fibril formation of peptides.^69,81,90,91^

If lipids were present at the same concentration as the peptide (1 : 1), Aβ_40_ aggregation was accelerated to a smaller extent compared to if lipids were present in an excess (1 : 9) (Fig. 2c–e). For U3.5, lower lipid molar ratios (peptide-to-lipid ratio 1 : 1) had either no effect on peptide aggregation, or, interestingly, aggregation was accelerated, particularly when POPG liposomes were present (ESI Fig. S3d†). When POPG or PazePC containing liposomes and micelles (POPG, PazePC, POPC-POPG, 4 : 1, and POPC-PazePC, 7 : 3) were present in excess (peptide-to-lipid molar ratio 1 : 9), U3.5 aggregation was completely inhibited (Fig. 2b, g, h and S3d, f, ESI†). Liposomes and micelles present large surface-to-volume ratios in the nanometre size range (ESI Fig. S2†).^61,92,93^ In the presence of strong electrostatic surface attraction, the adsorption of peptides onto micelles and liposomes can inhibit the formation of amyloid fibrils by depleting the free concentration of monomers in solution thereby reducing peptide mobility and flexibility. In the presence of weak surface attraction, micelle and liposome surfaces may act as potential adsorption and nucleation points for peptides and can seed aggregation.^8,39,94,95^ Similarly, if a small amount of surfaces with strong electrostatic attraction is present, surfaces can initiate the local concentration of peptide oligomers and thus accelerate peptide self-assembly.^17,92^ This may explain why U3.5 peptide aggregation was accelerated when POPG liposomes or PazePC micelles were present at low concentration (peptide-to-lipid molar ratio 1 : 1), but completely inhibited for ratios with lipid excess (1 : 9) (ESI Fig. S3d and f†). The change in peptide-to-lipid ratio is also related to a switch from peptide-rich to lipid-rich co-assemblies.^48,96^

Aβ_40_ peptide was influenced in a comparable manner by all lipids and lipid mixtures and thus a similar adsorption mechanism of the peptide to the liposomes is expected. While Aβ_40_ has an overall negative charge, it has both positively and negatively charged side chains as well as many hydrophobic residues that may all provide potential points of attraction to membrane surfaces. The importance of hydrophobic residues in Aβ_40_ for β-sheet formation has previously been demonstrated.^29,97^ In contrast, U3.5 peptide has an overall positive charge and thus attraction to negatively charged lipid headgroups is expected, leading to a strong influence of POPG and PazePC containing liposomes and micelles (POPG, PazePC, POPC-POPG, 4 : 1, and POPC-PazePC, 7.3). Uncharged POPC and cholesterol as well as POPC-cholesterol (4 : 1) liposomes did not significantly interact with U3.5 showing similar fluorescence profiles as without lipids present (Fig. 2f, S3b and h, ESI †). While lower POPG liposome amounts (peptide-to-lipid molar ratio 1 : 1) may have provided a nucleation point leading to acceleration (Fig. S3d†), the inhibition of U3.5 aggregation at a peptide-to-lipid molar ratio of 1 : 9 was likely caused by trapping of all the U3.5 monomers at the micelle and liposome surfaces. Thus, these fluorescence results indicate a competition between oligomer seed formation and the inhibition of aggregation through the binding of available peptide monomers to the membrane surface.

**Fig. 3:**
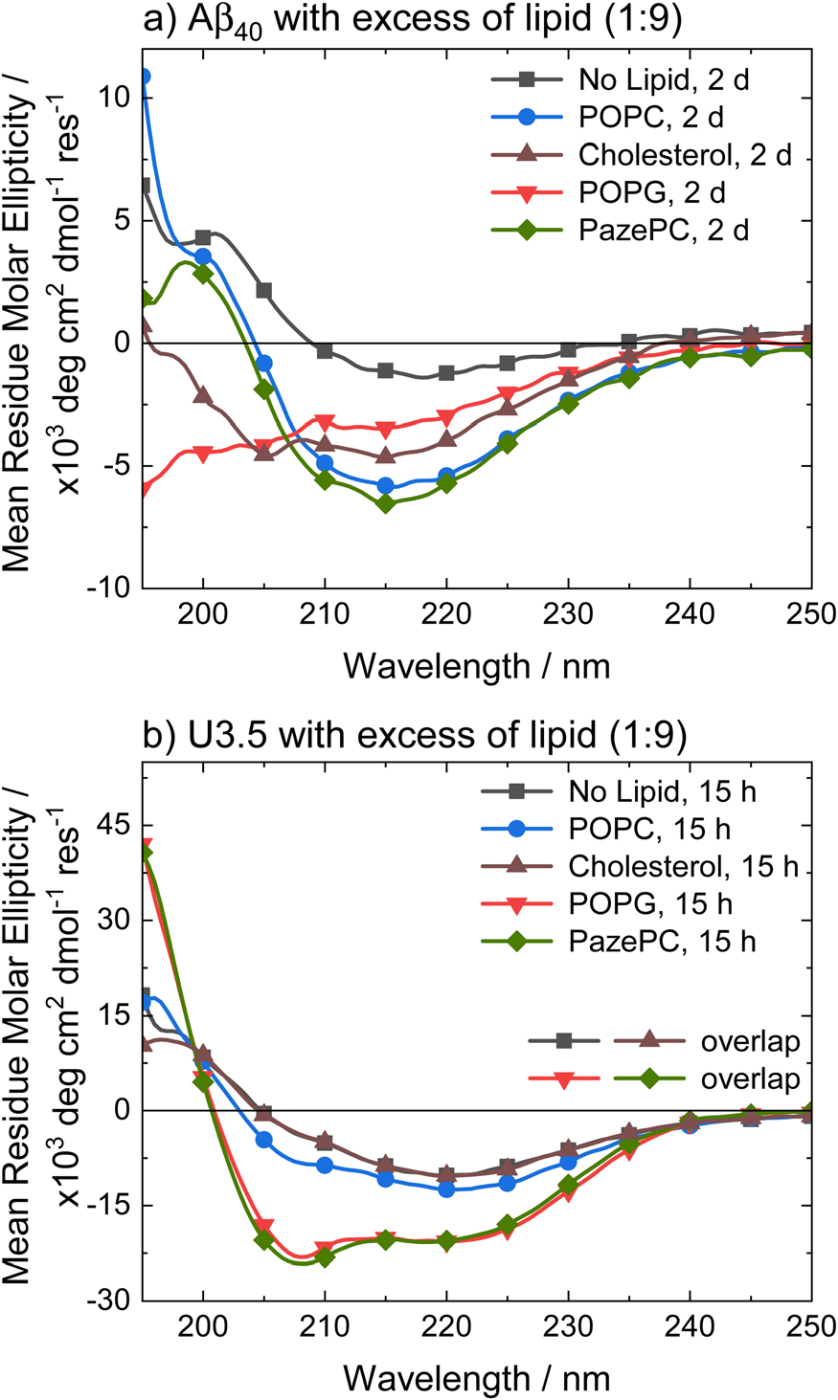
CD spectra of (a) Aβ_40_ and (b) U3.5 without and with excess of lipid (peptide-to-lipid molar ratio 1 : 9) in PBS buffer at pH 7.4 at 37 °C. Aβ_40_ aggregation was studied at 100 μM with 900 μM of lipid present (for CD, it was diluted to 20 μM peptide and 180 μM lipid) and U3.5 peptide was studied at 50 μM with 450 μM lipid present. Samples were measured after 2 days or 15 hours, respectively. Note that the data for U3.5 (b) without lipid present and with cholesterol were previously reported and are included here as reference to all other lipids and Aβ_40_.^17^ Note that the symbols are used to distinguish the data sets and data were recorded every 0.5 nm.

To better understand the distinct effects of the membrane components on peptide self-assembly, we studied peptide secondary structure changes of Aβ_40_ and U3.5 using circular dichroism (CD) spectroscopy (Fig. 3). The peptides were studied in the absence and presence of POPC, cholesterol, POPG and PazePC. The CD spectra show that the Aβ_40_ peptide aggregated and thus adapted a β-sheet conformation (*λ*_min_ at 215 nm) both in the absence and in the presence of lipids after two days of incubation. In contrast, U3.5 peptide showed β-sheet formation in the presence of POPC or cholesterol liposomes and without any lipid present (*λ*_min_ at 219 nm); however, the peptide was stabilized in an α-helical conformation (*λ*_min_ at 208 nm and 222 nm) when POPG liposomes or PazePC micelles were present;^98^ thus preventing β-sheet formation. The role of α-helical peptide conformations as potential intermediates towards fibrils and their high abundance at membrane surfaces has been discussed in the literature.^12,17,53^ It has previously been shown that peptides adopt a transmembrane conformation within membranes when the peptides are present in high concentration on the surface, resulting in an α-helical structure.^99^

Experiments with 2,2,2-trifluoroethanol (TFE) were performed to confirm that the solution environment has a distinct effect on Aβ_40_ secondary structure compared to U3.5. TFE is commonly used to enhance helical secondary structure.^17^ Our data show that while 40% TFE influenced the secondary structure of Aβ_40_ initially, the peptide aggregated after two days similarly to the Aβ_40_ samples with lipids (ESI Fig. S4†) (see Table S2† for quantitative secondary structure estimations from the experimental spectra). This is in contrast to U3.5 which was stabilized in its α-helical conformation for at least five days when 40% TFE was present, as previously reported.^100^ Bokvist *et al.* have shown that Aβ_40_ fibril formation is accelerated at membrane surfaces (for DMPC and DMPG) but prevented when the peptide was anchored in an α-helical conformation as a transmembrane peptide.^101^ In our study, we added liposomes and micelles to the peptides and thus Aβ_40_ was exposed to membrane surfaces and an accelerating effect expected.

**Fig. 4:**
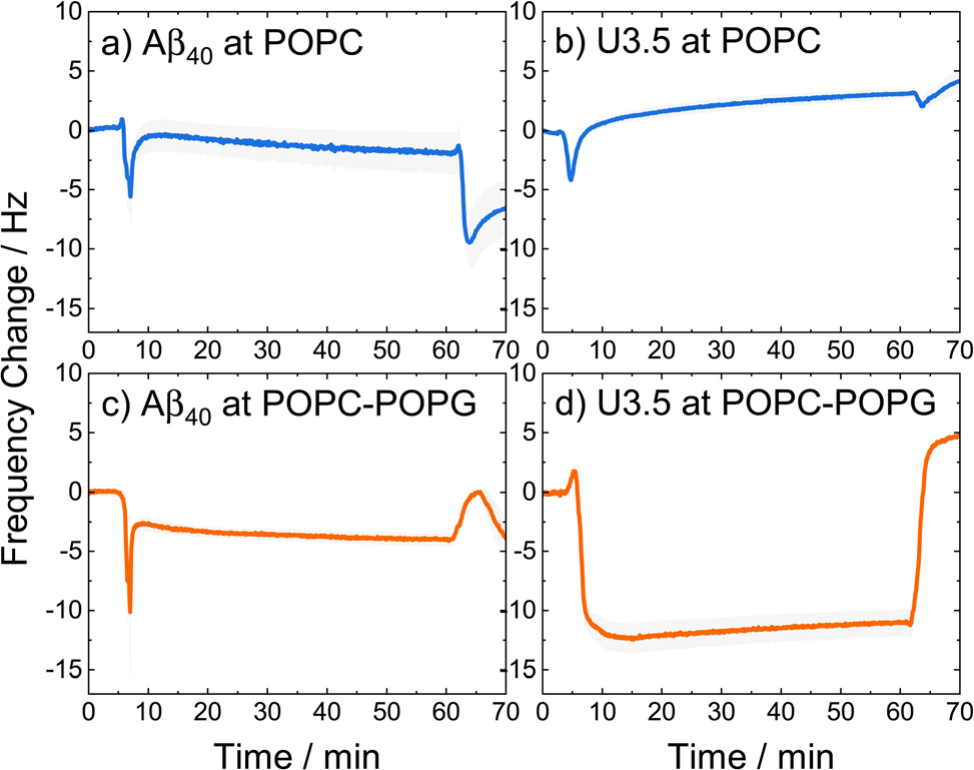
QCM changes in frequency of (a and c) Aβ_40_ and (b, d) U3.5 peptide (25 μM) interacting with (a and b) POPC and (c and d) POPC-POPG (4 : 1) lipid bilayers in PBS buffer at pH 7.4 at 22 °C. The larger the negative change in frequency, the stronger the peptide mass binding to the membrane. A lipid bilayer is first deposited on the sensor surface before the peptide is introduced (0–15 min), kept incubating (15–60 min), and finally rinsed with buffer (60–70 min). The solid lines refer to the mean and the shadow areas to the SEM (standard error of the mean) of the replicates.

The stabilization of U3.5 in an α-helical conformation in the presence of excess POPG liposomes or PazePC micelles (Fig. 3) is thus linked to the inhibitory effects on peptide aggregation observed in the ThT assays (ESI Fig. S3d and f†), while an intermediate stabilization may accelerate peptide self-assembly. Since POPG and partially PazePC lipids are negatively charged and U3.5 positively charged, it seems that electrostatic attraction and thus strong adsorption of U3.5 to the membrane surface could be the cause for the inhibitory effects on fibril formation while stabilizing the peptide in an α-helical conformation. To probe differences in peptide-membrane binding, we performed quartz crystal microbalance (QCM) measurements (Fig. 4). This technique enables monitoring of nanogram binding events to membrane surfaces using a surface-modified quartz crystal sensor.^102–105^

The QCM curves show a transient decrease in frequency and thus transient peptide binding for both peptides when interacting with POPC lipid bilayers (around 5 min), while only Aβ_40_ shows this behaviour when interacting with POPC-POPG (4 : 1). In contrast, the U3.5 peptide remained bound within the POPC-POPG membrane over time until the measurement cell was rinsed with buffer. It can also be seen that both peptides showed greater mass binding with the POPC-POPG lipid bilayers (Fig. 4c and d) compared to pure POPC (Fig. 4 and b). While the peptide-membrane interaction mechanism requires a more detailed study for its elucidation, we used QCM measurements here for the purpose to probe differences in interaction affinity of the peptides to the membrane surfaces. Our results confirm the hypothesis that the peptides bind more strongly to negatively charged lipids, shown here for POPC-POPG due to its stability in liposome and membrane formation, and particularly true for the net positively charged U3.5 peptide.

**Fig. 5:**
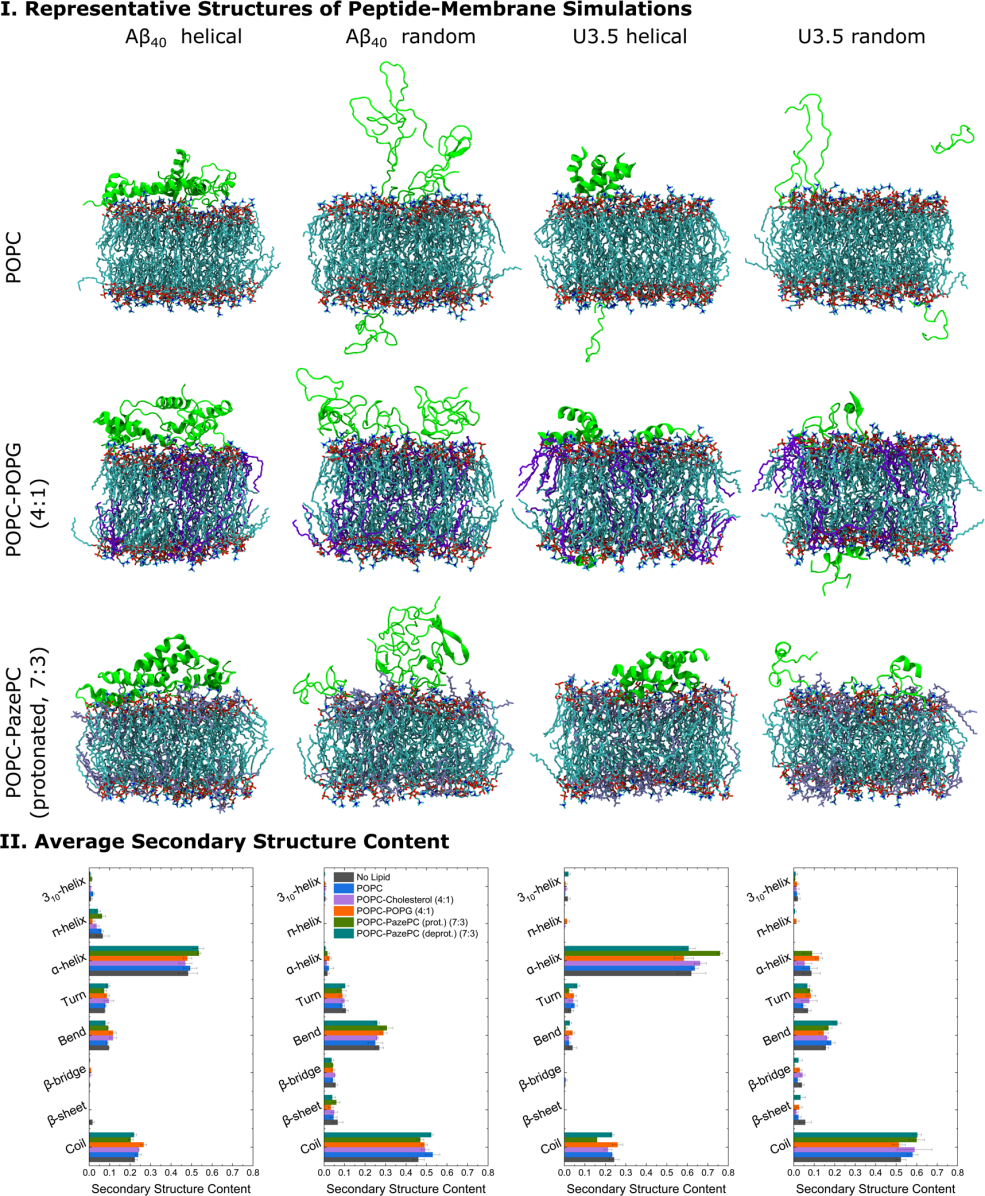
(I) Representative structures of the peptide-membrane simulations with five peptide monomers at 303.15 K and with 0.15 mM NaCl in water. The central structure of the largest structural cluster during the last 10 ns simulation time of all replicates for each peptide is shown when studied near POPC, POPC-POPG (4 : 1), and POPC-PazePC (protonated) (7 : 3) membranes. (II) The average secondary structure content of the peptides during the last 10 ns simulation time of all replicates is shown. MD simulation snapshots were visualized in VMD 1.93.^112^ Note that we studied membranes containing both protonated as well as deprotonated PazePC due to the potential presence of both under experimental conditions (pH 7.4).

Molecular dynamics (MD) simulations were performed to obtain detailed insight into peptide-membrane interactions.^78,106–108^ Both Aβ_40_ and U3.5 adsorbed to the lipid membranes within a few nanoseconds of simulation time; however, with differences depending on the peptide, initial secondary structure and membrane composition. Aβ_40_ and U3.5 were studied with both an α-helical and unstructured (random coil) initial structure since many peptides are unstructured in solution and tend to form helices near membranes.^17,109^ Moreover, our atomistic MD simulations can only sample limited time scales and we thus considered both conformations as starting structures. Cases with one and five peptide monomers were studied to understand binding and oligomer formation. Representative snapshots of the most dominant structures of the simulations show a strong adsorption of the peptides with an α-helical initial conformation to the membrane surface (Fig. 5 and S6–S10†, ESI). While Aβ_40_ adsorption to the membranes was comparable for all membranes, U3.5 bound more strongly to POPG and PazePC containing membranes than to pure POPC or Cholesterol containing membranes. Near POPC and POPC-Cholesterol membranes, U3.5 formed bundles of α-helices, similar to the simulations in solution without lipids present (ESI Fig. 5†). These helical bundles may be important oligomeric species in the pathways towards fibrils. When POPG or PazePC were present, U3.5 helices bound in their entire length to the membrane surface, preventing oligomer formation. Peptide-membrane adsorption appears particularly tight for the U3.5 peptide near POPC-POPG (4 : 1) and POPC-PazePC (7 : 3) membranes (see Fig. 5 and S6–S14, ESI† for simulation snapshots and mass density profiles). The tighter membrane binding of U3.5 compared to Aβ_40_ to POPG and PazePC containing membranes is consistent with our experimental QCM results (Fig. 4) and is likely related to stronger electrostatic interactions, as it has been previously demonstrated for different charged surfaces.^100^ This agrees with our observation that fibril formation was completely inhibited for U3.5 peptide when POPG or PazePC containing membranes were present in excess (Fig. 2).

When analysing the secondary structure of the peptide conformations in the simulations (Fig. 5 and S15, ESI†), we observed that the α-helical starting structure remained stable overall for both peptides with higher helical contents for U3.5 peptide. A greater β-sheet/bridge formation was found when the initial peptide structure was random coil. Significantly, our MD simulations show a stronger stabilization of the initially unstructured U3.5 peptide in an α-helical conformation than in a β-sheet/bridge, conforming the different impact of the studied membranes on both Aβ_40_ and U3.5. The MD simulations are in overall agreement with our experimental observations with a tighter membrane binding and secondary structure stabilization (Fig. 5) and thus larger influence on peptide aggregation for the U3.5 peptide. The relevance of the environmental conditions on the U3.5 peptide conformation has recently also been reported by Landau *et al.* who have determined a cross-α helical structure when in an environment of a polyether based on polypropylene glycol,^12^ and a cross-β fibril structure in aqueous solution.^110^ Previous studies also found that the related Aβ_42_ peptide is influenced in oligomer structure formation depending on the membrane composition and its environment.^111^ An accelerated accumulation of β-sheet secondary structure was observed on membranes containing oxidatively damaged phospholipids.^69^

**Fig. 6:**
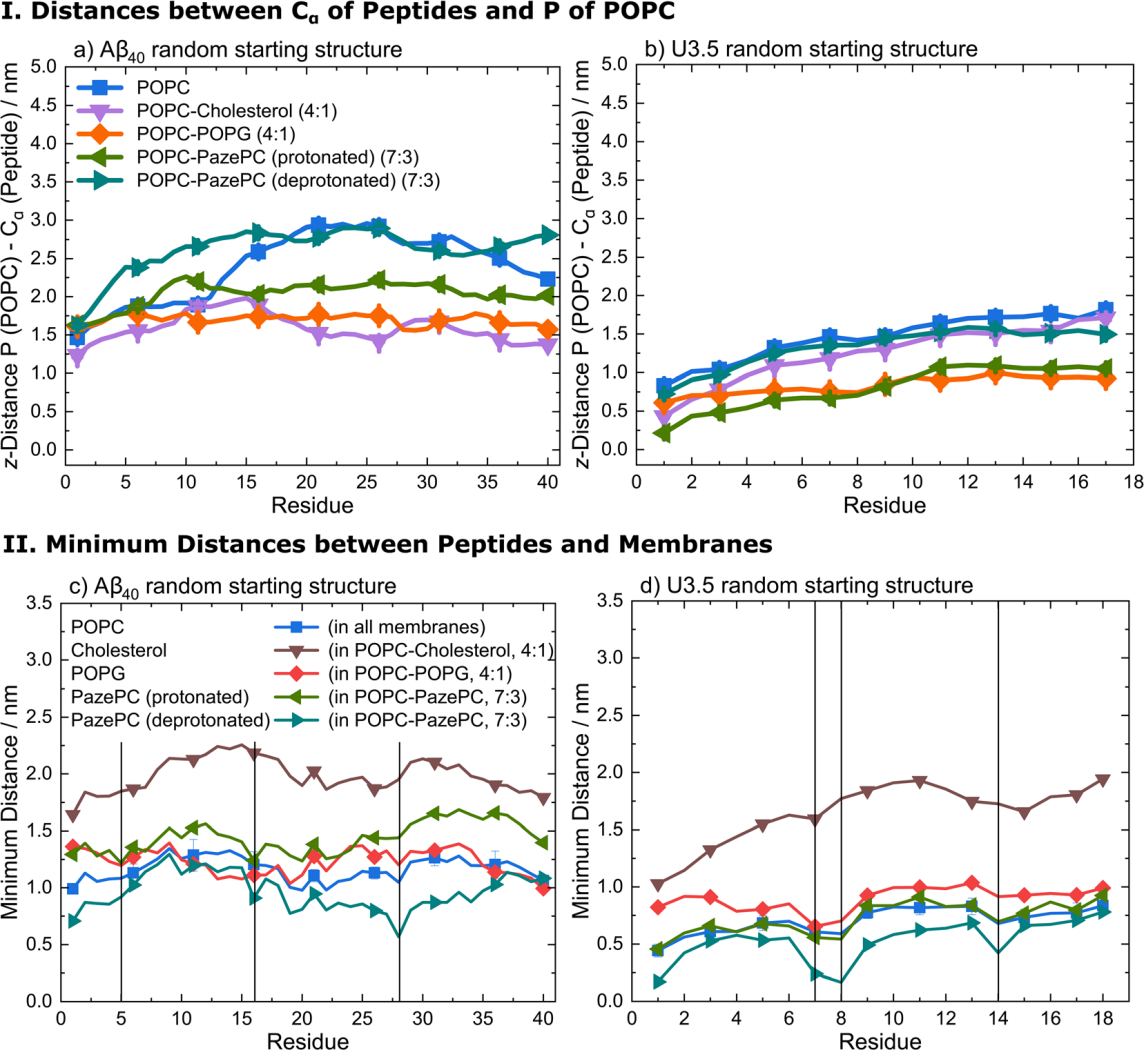
(I) Average distances between the phosphate head groups of POPC in the outer membrane leaflet and the peptide C_α_ atoms of (a) Aβ_40_ and (b) U3.5 with random starting structures (perpendicular to the membrane along *z*-axis) during the last 10 ns simulation time of all replicates. (II) Average minimum distances between the peptides (c) Aβ_40_ and (d) U3.5 with random starting structures and the lipid bilayer components POPC, cholesterol, POPG, and PazePC during the last 10 ns simulation time of all replicates. The vertical lines at residues 5 (arginine), 16 (lysine) and 28 (lysine) for Aβ_40_ and at residues 7 (arginine), 8 (arginine) and 14 (lysine) for U3.5 indicate the positively charged residues in both peptides to guide identifying the closest peptide-membrane interactions. Note that the symbols are used to distinguish the data sets and each residue has a data point.

Analysis of the average distances between the phosphate headgroups of POPC in the outer membrane leaflet and the peptide C_α_ atoms confirmed the tighter binding of U3.5 peptide to POPG and PazePC containing membranes (Fig. 6 and S16, ESI†). Further analysis of the closest interactions between the peptides and the membrane components confirmed our suggestion that differences in the charge between both peptides may cause the differential impact caused by POPC, anionic POPG and oxidized PazePC lipids (Fig. 6 and S17, ESI†). The cationic amino acids arginine and lysine in positions 5, 16 and 28 in Aβ_40_, and in positions 7, 8 and 14 in U3.5 showed minima in peptide-membrane distance, and thus indicate the most dominant peptide-membrane interactions. Electrostatic attraction was particularly observed for the U3.5 peptide. In previous work, we already demonstrated the high relevance of position 7 (arginine) in U3.5 for peptide aggregation and membrane interactions.^8,17^ Our MD simulations show that positions 8 and 14 were of high relevance for the initial membrane interactions for the U3.5 peptide, for both the simulations with an α-helical and random starting structure. We note that longer simulation time scales would be required to study more detailed effects on the membrane integrity. However, our study here focused on the effects on peptide adsorption and self-assembly.

**Fig. 7:**
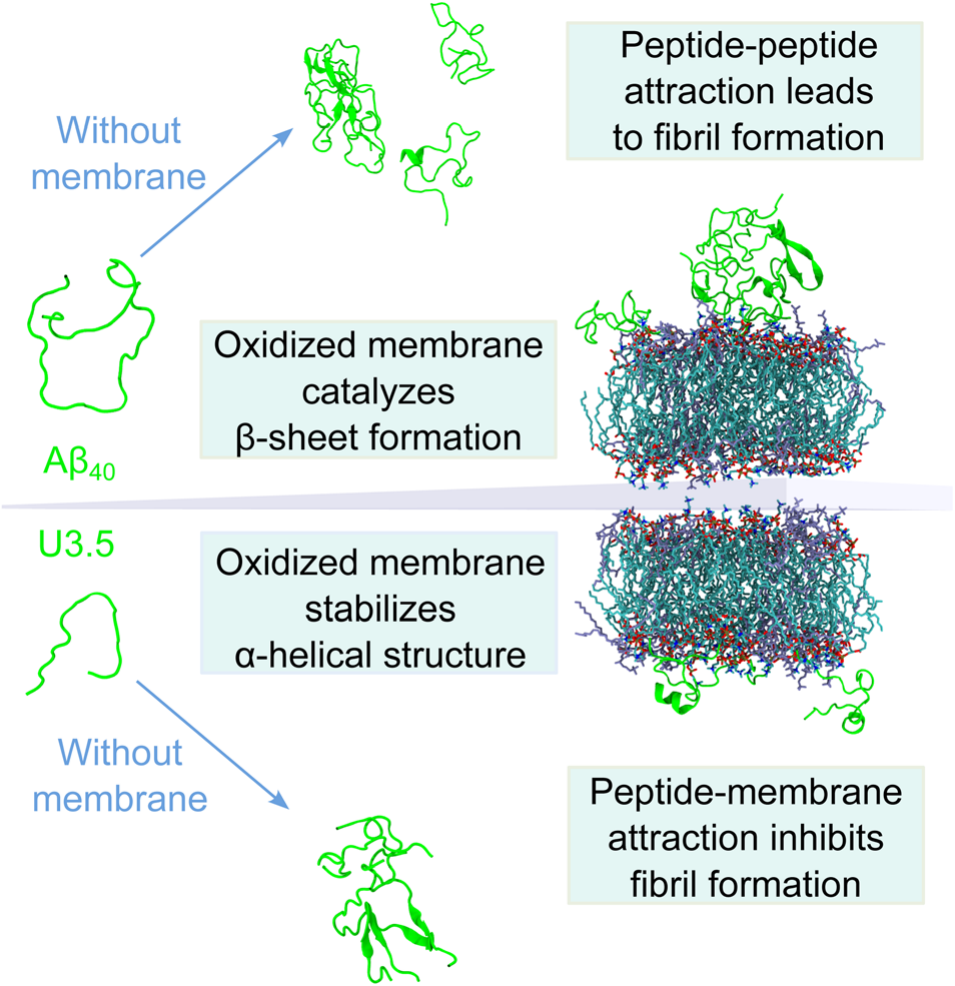
Model illustrating the differential impact of (oxidized) membranes on peptide self-assembly into amyloid fibrils. While both Aβ_40_ and U3.5 form β-sheet rich structures in solution without membranes, oxidized membranes accelerated Aβ_40_ aggregation, while U3.5 aggregation was inhibited when lipids were present in excess. This was driven by stronger peptide-membrane attraction and α-helical stabilization, whereas peptide–peptide interactions drive self-assembly into fibrils. A balance between peptide aggregation propensity and surface attraction determines the fate of peptide self-assembly; thus small changes in lipid composition can alter membrane impact.

In summary, our data have shown that membrane lipid composition and particularly lipid oxidation modulate the effect of membranes on peptide oligomerization and self-assembly (Fig. 7). This is in agreement with previous studies that have shown that membrane composition can both lead to inhibition and enhancement of peptide aggregation.^40^ For the overall negatively charged and highly hydrophobic Aβ_40_ peptide, peptide aggregation was accelerated by all membranes at the peptide-to-lipid ratios that we assessed, especially by POPC and PazePC containing membranes. In contrast, the net positively charged U3.5 peptide was not significantly influenced by POPC or cholesterol but showed some acceleration at low amounts of POPG and PazePC containing membranes, and complete inhibition when POPG and PazePC lipids were present in excess. Habchi *et al.* have previously demonstrated that cholesterol containing membranes can accelerate Aβ_42_ aggregation through a heterogeneous nucleation pathway.^65^ Krausser *et al.* showed that high membrane fluidity increases the rate of peptide aggregation by allowing lipids to be incorporated into the fibrils.^39^ Our QCM experiments and MD simulations confirmed a strong binding of U3.5 to POPG and PazePC containing membranes, with stabilization of the peptide in the α-helical conformation, revealed by CD spectroscopy and MD simulations. Our work demonstrates computationally the existing hypothesis that the oxidized lipid PazePC can inhibit peptide aggregation by stabilization of the peptide in an α-helical conformation.^81^ The impact of membrane lipids on peptide aggregation, as well as changes in membrane composition, must therefore be peptide specific. The peptide sequence as well as the peptide aggregation propensity are important factors next to the peptide-membrane surface attraction.^113^ The membrane composition, influenced by oxidation processes, results in distinct physical properties of the membranes,^77,114–117^ which in turn impact the effects on peptide aggregation.^118^ In our study, we have investigated the effects for the net negatively charged Aβ_40_ peptide and the net positively charged U3.5 peptide. Electrostatic interactions mediated by the presence of charged peptide residues were highly relevant for the U3.5 peptide, while Aβ_40_ peptide experienced intermediate affinity to all membranes. This is in agreement with previous work that identified electrostatic interactions to be particularly important for cationic peptides and hydrophobic peptide residues to drive the interactions of nonpolar residues with membranes.^66,119,120^ The sequence and number of positively charged residues in peptides, such as arginine and lysine, may thus have important influence on the degree and direction in which anionic and oxidized lipids affect peptide aggregation.

## Conclusions

This work suggests the importance of (cellular) lipid membranes, particularly their biochemical composition, for biologically relevant processes, such as peptide adsorption, secondary structure stabilization and aggregation. Changes in the membrane structure due to oxidative reactions or the infection with bacteria and thus the exposure to new and distinctly modified cell surfaces may lead to enhanced peptide adsorption to membrane surfaces, resulting in the acceleration or inhibition of peptide aggregation, and in turn amyloid fibril formation as shown in our work. It seems obvious that these effects may have implications for the link between amyloidogenic peptides and the development of neurodegenerative diseases. The strong effect of the anionic, bacteria-mimicking, and oxidized membrane lipids on the antimicrobial peptide U3.5 emphasize our hypotheses about existing links between antimicrobial and amyloidogenic peptides, as well as to infection and (oxidative) stress. Both Aβ_40_ and U3.5 may have a functional role in organisms and their self-assembly into α-helical or β-sheet rich conformations be linked to functional or disease-related states. Changes in the cellular membrane and peptide conformation due to stress could thus trigger functional loss of peptides and proteins.

## Abbreviations

Aβ_40_: amyloid β (1–40)
AMP: antimicrobial peptide
CD: circular dichroism
CMC: critical micelle concentration
DLS: dynamic light scattering
DMPG: 1,2-dimyristoyl-*sn*-glycero-3-phospho-(1′-rac-glycerol)
MD: molecular dynamics
PazePC: 1-palmitoyl-2-azelaoyl-*sn*-glycero-3-phosphocholine
PBS: phosphate-buffered saline
POPC: 1-palmitoyl-2-oleoyl-*sn*-glycero-3-phosphocholine
POPG: 1-palmitoyl-2-oleoyl-*sn*-glycero-3-phospho-(1′-rac-glycerol)
QCM: quartz crystal microbalance
TFE: 2,2,2-trifluoroethanol
ThT: thioflavin T
U3.5: uperin 3.5

## Data availability

All data needed to evaluate the conclusions of this study are presented in the paper or the ESI.† In addition, data are available on Zenodo at https://doi.org/10.5281/zenodo.7665947.

## Author contributions

The study was designed and conceptualized by TJ, BA, HJR and LLM. Biophysical studies were performed and analysed by TJ (ThT fluorescence assays, CD spectroscopy, DLS measurements), SP (QCM and CD measurements) and TD (CD spectroscopy). Computer simulations were performed and analysed by TJ. The results were discussed and interpreted by TJ, BA, HRJ and LLM. The manuscript was written by TJ and advanced by all authors.

## Conflicts of interest

There are no conflicts to declare.

## Supplementary Material

SC-014-D3SC00159H-s001
